# Association between chromosome 22q11.2 translocation and male oligozoospermia

**DOI:** 10.1097/MD.0000000000030790

**Published:** 2022-09-30

**Authors:** Peng Zhan, Tingting Hao, Xiao Yang, Yi Zhang

**Affiliations:** a Department of Urology, the Second Hospital of Jilin University, Changchun, China.

**Keywords:** chromosome 22, genetic counseling, oligozoospermia, translocation

## Abstract

Chromosomal aberrations in peripheral blood are a major cause of reproductive disorders for the infertile couples. Reciprocal translocation is closely related to male infertility. The breakpoint of translocation may disrupt or dysregulate important genes related to spermatogenesis. The relationship between some breakpoints of chromosome and male infertility has been paid attention. Chromosome 22q11.2 translocation has not been reported with male infertility. The purpose of this study is to evaluate the relationship between chromosome 22q11.2 translocation and male infertility. All patients were collected from the second hospital of Jilin University. Semen parameters were detected using the computer-aided semen analysis system. Cytogenetic analysis was performed using standard operating procedure. Related genes on chromosomal breakpoints were searched using online mendelian inheritance in man (OMIM). The association between this breakpoint and spermatogenesis is also discussed. We report 6 cases of translocation in chromosome 22. Of 7 breakpoints involved in these translocations, the common feature is that they all included chromosome 22q11.2 translocation and presented with oligozoospermia. The analysis of breakpoint related genes showed testis-specific serine/threonine kinase 2 (*TSSK2*) gene is associated with human spermatogenesis impairment. Overall, these results suggest that the breakpoint involved in translocation deserves attention from physicians in genetic counseling. The breakpoint rearrangement has the possibility of disrupting spermatogenesis. The relationship between 22q11.2 breakpoint and male infertility deserves further study.

## 1. Introduction

Male infertility is a common clinical problem in urological practice, and is a pathological condition with a genetic background.^[1]^ Chromosomal aberration is reported to one of the common causes in infertile men,^[2]^ and is detected in 14% of infertile patients.^[3]^ Reciprocal balanced translocation is one of the most frequently occurring human chromosomal abnormalities.^[4]^ Due to the limitations of classical G-banding analysis, the incidence of reciprocal translocation is often underestimated.^[5]^ It has been reported that individuals with reciprocal balanced translocation easily exhibit azoospermia or oligozoospermia.^[6]^ However, male translocation carriers with normal fertility are often found in clinical practice. Hence, Genetic counseling is still challenging for these patients.

The specific mechanisms underlying the effects of chromosomal translocation on fertility remains unclear for the majority of carriers.^[5]^ A large number of studies showed that balanced translocation may reduce fertility due to the production of unbalanced gametes.^[7–10]^ Some studies showed that the breakpoint of chromosome translocation may disrupt or dysregulate important genes related to spermatogenesis, which lead to infertility.^[7,11]^ The relationship between some breakpoints of chromosome and male infertility has been reported. Singh et al^[12]^ reported that 19p13.3 duplication is associated with severe testicular phenotypes of infertile men. Li et al^[13]^ reported that chromosome 1q21 translocation is closely related to azoospermia. Zhang et al^[14]^ reported that the breakpoints at 10p12 and 10q26.3 are associated with azoospermia or oligozoospermia.

This study reported 6 males with chromosome 22q11.2 translocation. Moreover, the association between breakpoint 22q11.2 and male oligozoospermia has been discussed considering published cases as well.

## 2. Materials and Methods

This study was approved by the Ethics Committee of the Second Hospital, Jilin University. Written informed consent has been obtained from all participants for the publication of these cases.

### 2.1. Patients

All patients included here had visited the andrology outpatient department of the Second Hospital, Jilin University, China. A questionnaire survey was conducted to collect patient data, such as age, marriage status, pregnancy history, genetic family history, anamnesis information, smoking and drinking history, and intervention of drugs. Physical examination was performed to record patients’ height, weight, growth and development information, and testicular size.

### 2.2. Semen analysis

After abstinence for 3 to 7 days, patients’ semen was collected in a sterile container and examined by 2 professional technicians after liquefaction. Semen parameters were detected using the computer-aided semen analysis system (Beion S3, Shanghai Beion Medical Technology Co., Ltd, Shanghai, China). Oligozoospermia was diagnosed when sperm concentration was lower than the reference value of 15 × 10^6^ per mL.

### 2.3. Cytogenetic analysis

Peripheral blood (2 mL) was collected from all patients in sterile tubes containing heparin anticoagulant. Lymphocytes were cultured in RPMI-1640culture medium (including phytohemagglutinin) for 72 hours. Then, G-banding was performed using standard operating procedure. At least 20 metaphases were analyzed for each patient. The karyotypes were described according to the International System for Human Cytogenetic Nomenclature (ISCN 2016).

### 2.4. Analysis of related genes

To explore the relationship between translocation breakpoints and clinical phenotype, related genes on these breakpoints were searched using Online Mendelian Inheritance in Man (OMIM; https://www.ncbi.nlm.nih.gov/omim).

## 3. Results

### 3.1. Patient characteristics and clinical presentation

**Figure 1.**
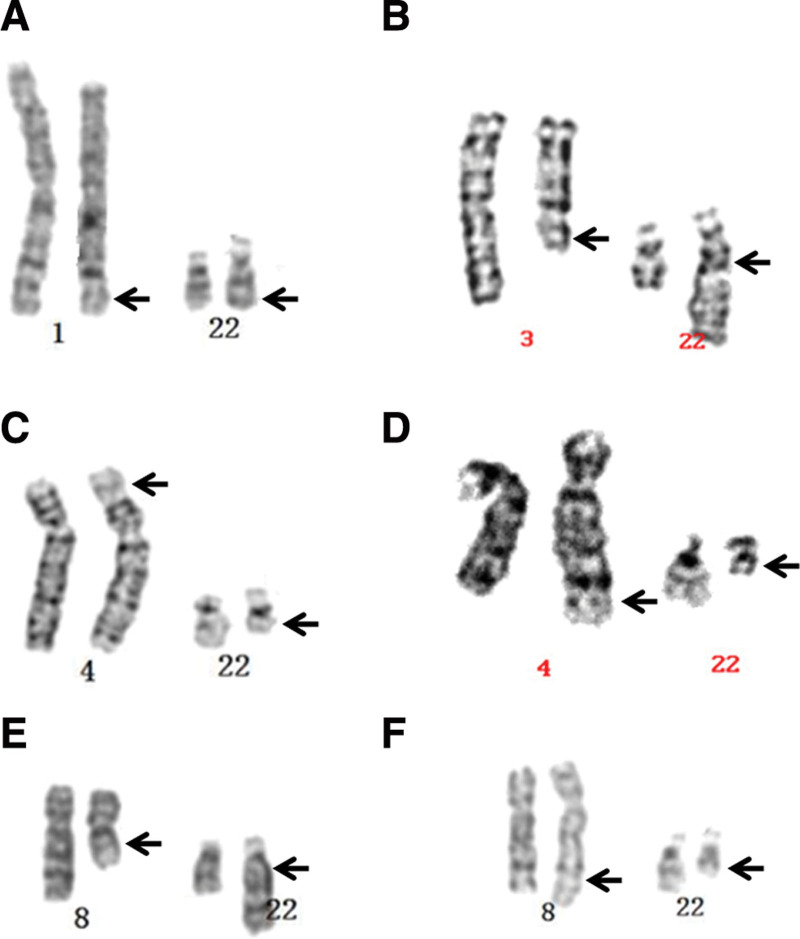
G-banding karyotypes of 6 patients in this study.

The subjects of this study were 6 male carriers of chromosome translocation. The clinical features of all patients were oligozoospermia. The clinical findings and Karyotypes of these patients are collected in Table 1. The common feature of the 6 patients was that they all included chromosome 22q11.2 translocation. The karyotype diagram is shown in Figure 1.

**Table 1:** Clinical findings and Karyotype of the subjects of this study.

Cases	Age	Clinical findings	Karyotype	Figure
1	30	Oligoasthenospermia	46,XY,t(1;22)(q32;q11.2)	1(A)
2	31	Oligozoospermia	46,XY,t(3;22)(q12;q11.2)	1(B)
3	32	Oligoasthenoteratozoospermia	46,XY,t(4;22)(p16;q11.2)	1(C)
4	36	Oligozoospermia	46,XY,t(4;22)(q35;q11.2)	1(D)
5	28	Oligoteratozoospermia	46,XY,t(8;22)(q13;q11.2)	1(E)
6	31	Oligoasthenospermia	46,XY,t(8;22)(q24;q11.2)	1(F)

### 3.2. Translocation breakpoint analysis

Seven breakpoints (1q32, 3q12, 4p16, 4q35, 8q13, 8q24, and 22q11.2) were involved in these translocations. Related genes and functions at the translocation breakpoints were collected in Table 2. Of these genes, testis-specific serine/threonine kinase 2 (*TSSK2*) gene is associated with human spermatogenesis impairment.

**Table 2:** Related genes and functions at translocation breakpoints of this study.

Breakpoint	Gene	Full name of gene	Function	
1q32	*ATP2B4* (108732)	ATPase, Ca^2+^-transporting, plasma membrane,4	Related to sperm motility	Okunade et al^[15]^
	*ADORA1*(102775)	Adenosine A1 receptor	Play a role in fertilization process	Allegrucciet al.^[16]^
3q12	N/A	N/A	N/A	
4p16	N/A	N/A	N/A	
4q35	*CFAP97* (616047)	Cilia-and flagella-associated protein 97	CFAP97 is highly expressed in adult testis, and is predicted to be related to the assembly and/or stability of motile cilia	Nagase et al^[17]^
8q13	N/A	N/A	N/A	
8q24	*HSF1*(140580)	Heat-shock transcription factor 1	Highly and specifically expressed in nuclei of spermatocytes and round spermatids	Akerfeltet al.^[18]^
22q11.2	*TSSK2*(610710)	Testis-specific serine/threonine kinase 2	Be associated with human spermatogenesis impairment	Zhang et al^[19]^

N/A = not applicable.

## 4. Discussion

Chromosomal translocations are a significant chromosomal structural abnormality,^[6]^ and are well-known causes of reproductive failure.^[5]^ Most of male carriers involved in sex chromosome translocation show azoospermia.^[20–23]^ About 60% of male carriers with autosomal translocation have at least one abnormal parameter in their semen analysis.^[24,25]^ The difference of these semen parameters depends on the specific chromosome and breakpoints involved in translocation.^[5,24,26]^

This study reports 6 cases of male carriers with chromosome 22 translocation. Chromosome 22 rearrangements have been reported to be associated with male or female fertility. Jaillard et al^[27]^ reported that 22q11.2 rearrangement is associated with low ovarian reserve and premature ovarian insufficiency in women. Özcan et al^[28]^ reported that a case of 22q11.2 deletion syndrome with azoospermia, and speculated that azoospermia can be one of the unknown clinical features of this syndrome. Chakraborty et al^[10]^ reported a case of 46,XY,t(19;22)(19q13.4;22q11.2) with azoospermia. Gada Saxena et al^[29]^ reported that a case of 46,XY,t(11;22)(q23;q11) showed male infertility. Vegetti et al^[30]^ reported that a primary infertile patient with 46,XY,t(17;22) (q11;q11) presented with asthenoteratozoospermia.

Further analysis of the breakpoint of translocation shows that all 6 patients are chromosome 22q11.2. Meanwhile, the common clinical phenotype of these patients is oligozoospermia. We searched the related cases in the literature and explored the possible relationship between this breakpoint and spermatogenesis. Douet-Guilbert et al^[31]^ reported a case of 46,XY,t(9;22)(q21;q11.2) with oligozoospermia. Kuroda et al^[32]^ reported a case of 46,XY,t(2;22)(p16;q11.1) with oligozoospermia. Perrin et al^[33]^ reported a case of 46,XY,t(9;22)(q21;q11.2) with oligoasthenospermia. The coexistence of chromosome 22q11.2 translocation and oligozoospermia may be a coincidental, but one of the unknown relationships between this breakpoint and oligozoospermia may exist.

A chromosome rearrangement may disrupt or dysregulate important genes related to spermatogenesis. Therefore, studying the relationship between chromosome abnormalities and clinical phenotype has become one approach to identifying genes involved in infertility.^[7]^ By using OMIM, *TSSK2* gene, which is located on chromosome 22q11.2, is associated with human spermatogenesis impairment. No genes related to spermatogenesis were found at the other chromosomal breakpoint of these translocations (1q32, 3q12, 4p16, 4q35, 8q13, 8q24). *TSSK2* gene may play an indispensable role in spermatogenesis process, and is associated with male idiopathic infertility in humans.^[19,34]^

One limitation of this study is the lack of further research regarding the specific genetic effects of this breakpoint by molecular-cytogenetic methods.

## 5. Conclusions

In conclusion, we report 6 male carriers of chromosome 22q11.2 translocation. This breakpoint rearrangement has the possibility of disrupting spermatogenesis, which can lead to oligozoospermia. The breakpoint should be assessed by physicians for male carriers in genetic counseling. The relationship between 22q11.2 breakpoint and male infertility deserves further study.

## Author contributions

**Conceptualization:** Peng Zhan, Xiao Yang.

**Data curation:** Tingting Hao, Xiao Yang.

**Investigation:** Peng Zhan, Tingting Hao, Xiao Yang.

**Methodology:** Yi Zhang.

**Writing – original draft:** Peng Zhan.

**Writing – review & editing:** Xiao Yang.
